# Primary Pulmonary Angiosarcoma Found Incidentally in a Complicated Patient: A Rare Case Report

**DOI:** 10.1111/crj.13818

**Published:** 2024-08-06

**Authors:** Reza Basiri, Alireza Ziaei Moghaddam, Arezoo Rikhtegar, Amir Hossein Jafarian

**Affiliations:** ^1^ Lung Disease Research Center, Faculty of Medicine Mashhad University of Medicine Sciences Mashhad Iran; ^2^ Faculty of Medicine Mashhad University of Medical Sciences Mashhad Iran; ^3^ Department of Internal Medicine, Faculty of Medicine Mashhad University of Medical Sciences Mashhad Iran; ^4^ Department of Pathology, Faculty of Medicine Mashhad University of Medical Sciences Mashhad Iran

**Keywords:** complicated patient, incidental finding of tumor, primary pulmonary angiosarcoma, rare case report

## Abstract

**Introduction:**

Primary pulmonary angiosarcoma (PPA) is a highly aggressive and rare malignancy originating from the endothelial cells of blood vessels in the lungs. PPA is an extremely rare subtype, with less than 30 cases reported to date. PPA is not only challenging to diagnose but also has a poor prognosis, often resulting in a high mortality rate within a year of diagnosis, regardless of the treatment approach.

**Method:**

We present the case of a 33‐year‐old woman with no significant past medical history who presented with abdominal pain and was incidentally found to have a right hilar mass with pleural effusion and empyema. After undergoing surgery for a perforated gastric ulcer, her pulmonary lesions were further worked up. Despite an extensive diagnostic evaluation, including imaging, bronchoscopy, and thoracotomy, establishing a diagnosis was challenging. Ultimately, PPA was diagnosed on surgical lung biopsy, and the patient was started on pazopanib and paclitaxel chemotherapy but expired after 1 month due to multiple complications.

**Conclusion:**

This case highlights the difficulty in diagnosing this rare tumor and its poor prognosis regardless of therapy. Greater awareness of PPA and more research are needed to improve early detection and treatment options for this deadly disease.

## Graphical abstract

This case report details the diagnostic complexity of primary pulmonary angiosarcoma (PPA), a rare and aggressive endothelial malignancy, found incidentally in a complicated 33‐year‐old woman, and serves to underscore the significant clinical challenges faced during the diagnostic process and the grim prognosis that accompanies this rare malignancy. Despite extensive investigative measures, the diagnosis remained elusive until postsurgical biopsy, following which the patient succumbed within a month.

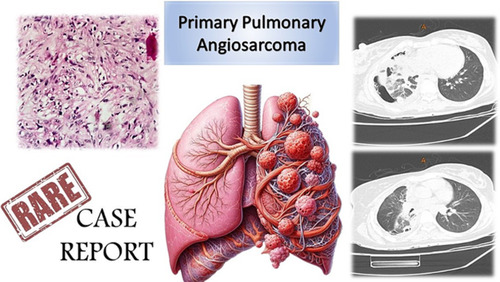

**Abbreviations**
BAL: bronchoalveolar lavage
CD: clusters of differentiation
CT: computed tomography
ICU: Internal Medicine Department's intensive care unit
IHC: immunohistochemistry
MPA: metastatic pulmonary angiosarcoma
NTM: nontuberculous mycobacterium
OR: operating room
PCR: polymerase chain reactions
PPA: primary pulmonary angiosarcoma
RLL: right lower lobe
SPECT/CT: single‐photon emission computed tomography
TBLB: transbronchial lung biopsy

## 1 Introduction

Angiosarcoma is a rare and aggressive tumor that originates from blood vessels or lymphatic vessels. It is a subtype of soft‐tissue sarcoma, representing approximately 2% of all cases. The worldwide incidence is estimated to be around 2 in 10 million. Angiosarcomas of the lung can be classified as primary pulmonary angiosarcoma (PPA) or metastatic pulmonary angiosarcoma (MPA) [1, 2].

Although pulmonary angiosarcomas have a rich vascular network, they are typically secondary tumors or MPA. Regarding PPA, less than 30 cases have been reported to date, based on a search of PubMed, especially MEDLINE [3, 4]. PPA is typically not diagnosed early due to its rarity and lack of case reports. A definitive diagnosis is usually based on histopathological and immunohistochemical findings [5]. We report a case of PPA of a young woman with right hemithorax mass plus pleural effusion and empyema, which was incidentally found on an abdominal and pelvic computed tomography (CT). After going through so many diagnostic challenges, we could diagnose by performing a thoracotomy and starting her treatment. Unfortunately, the patient was deceased after a month of her treatment.

## 2 Case Report

On August 10th, 2022, a 33‐year‐old nonsmoking housewife arrived at the Emergency Department with a sudden onset of worsening abdominal pain with nausea and vomiting.

**FIGURE 1:**
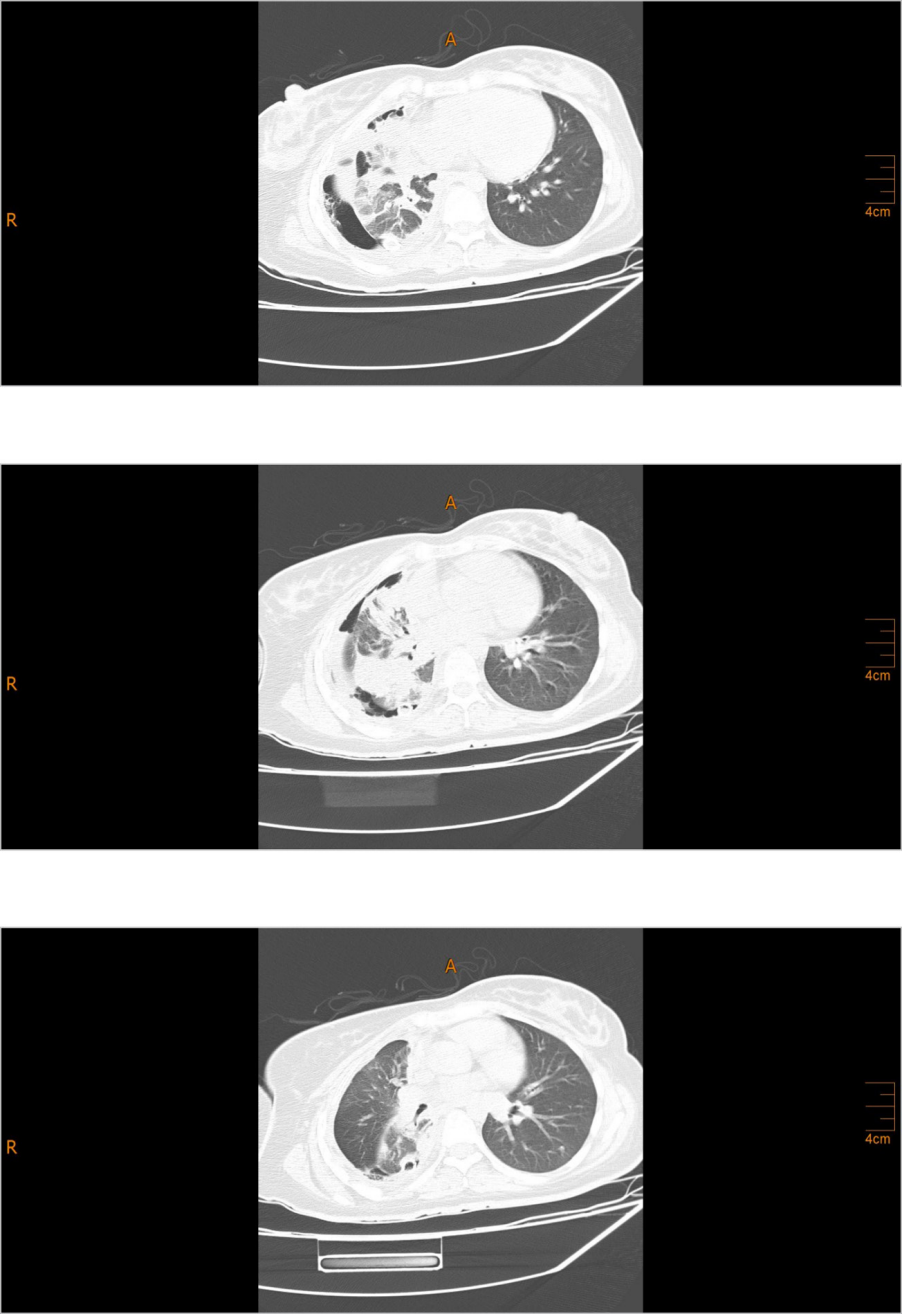
2022/04/17—spiral lung CT showing pleural effusion plus pyopneumothorax and consolidation causing the collapse of the RLL in the right hemithorax.

**FIGURE 2:**
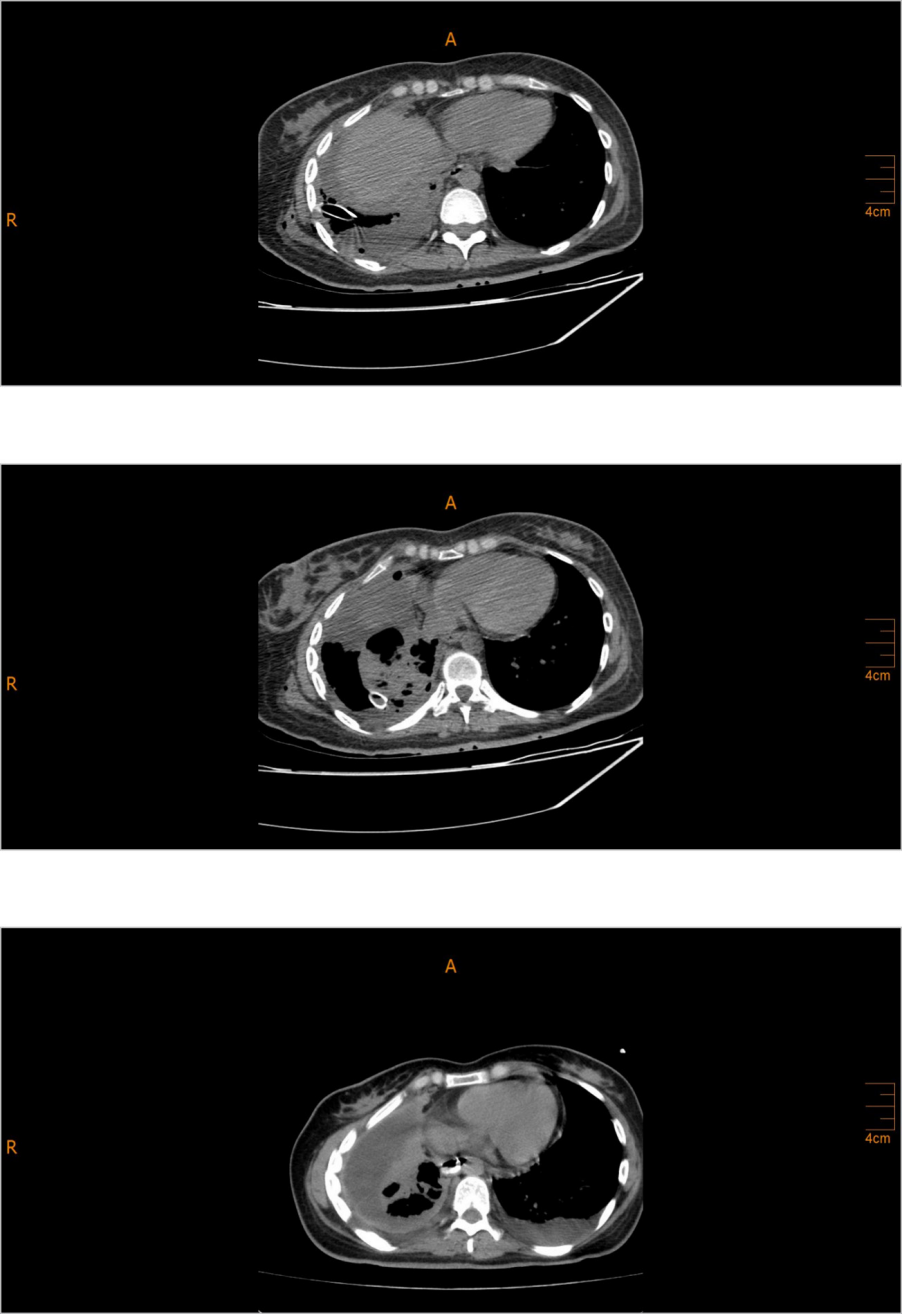
2022/08/10—spiral lung CT showing pleural effusion and a right perihilar consolidation causing the collapse of the RLL in the right hemithorax.

**FIGURE 3:**
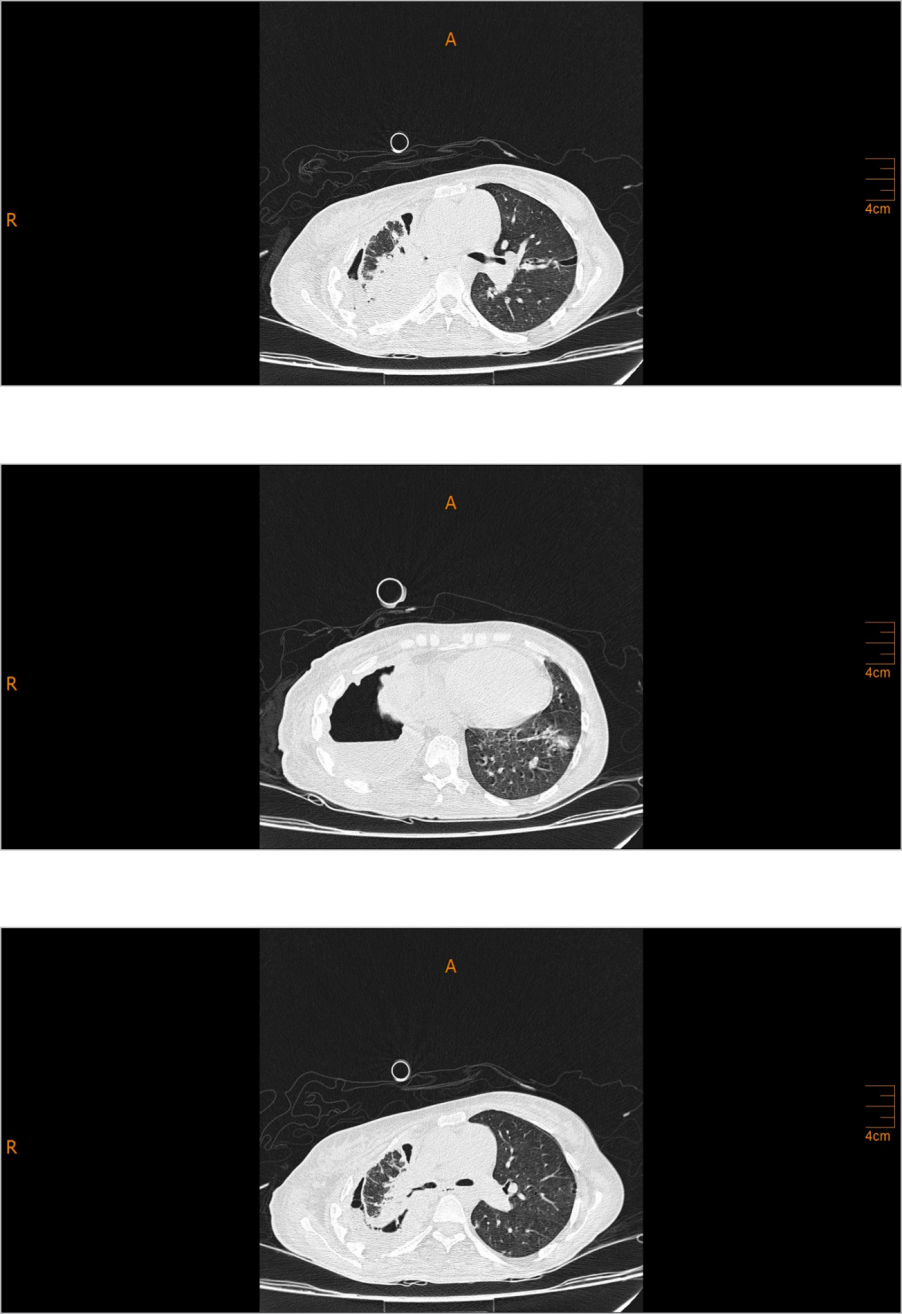
2022/08/10—spiral lung CT after surgery showing pleural effusion plus pyopneumothorax and consolidation causing the collapse of the RLL in the right hemithorax.

**FIGURE 4:**
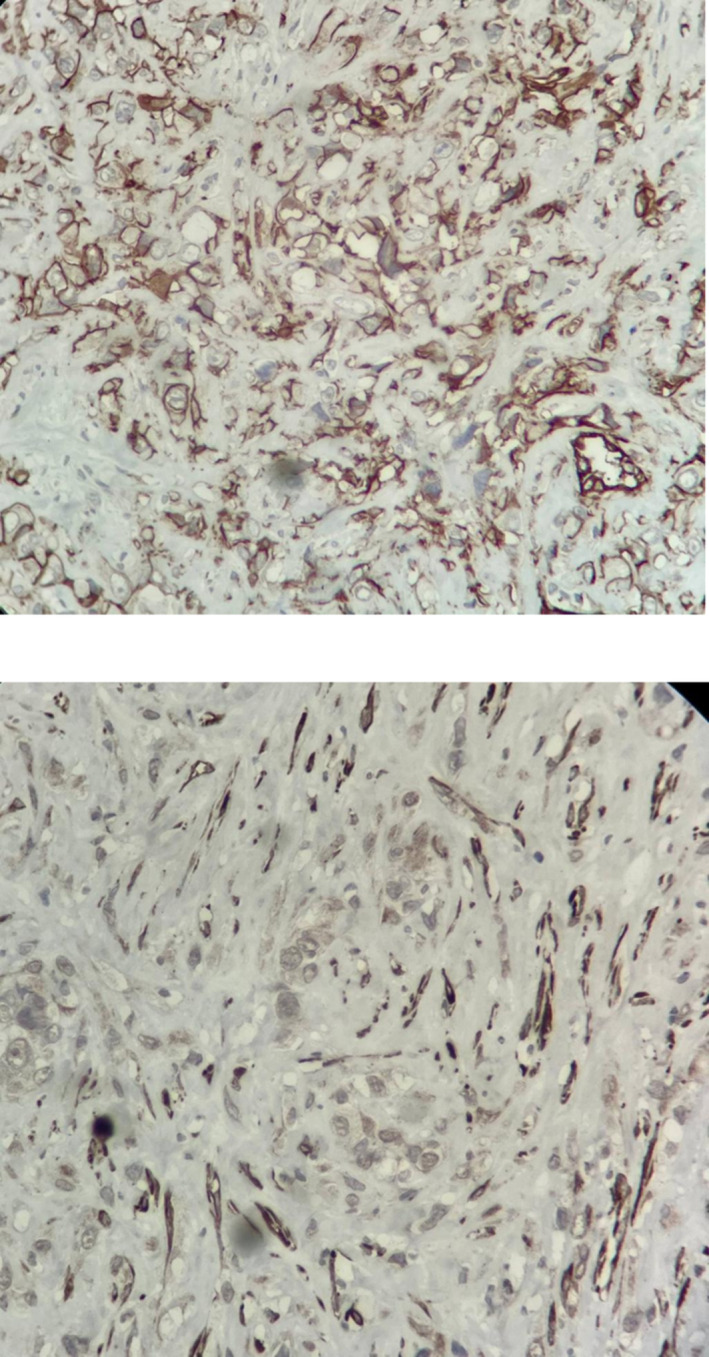
IHC revealing positive CD31 marker and negative cytokeratin staining, respectively, 100×.

**FIGURE 5:**
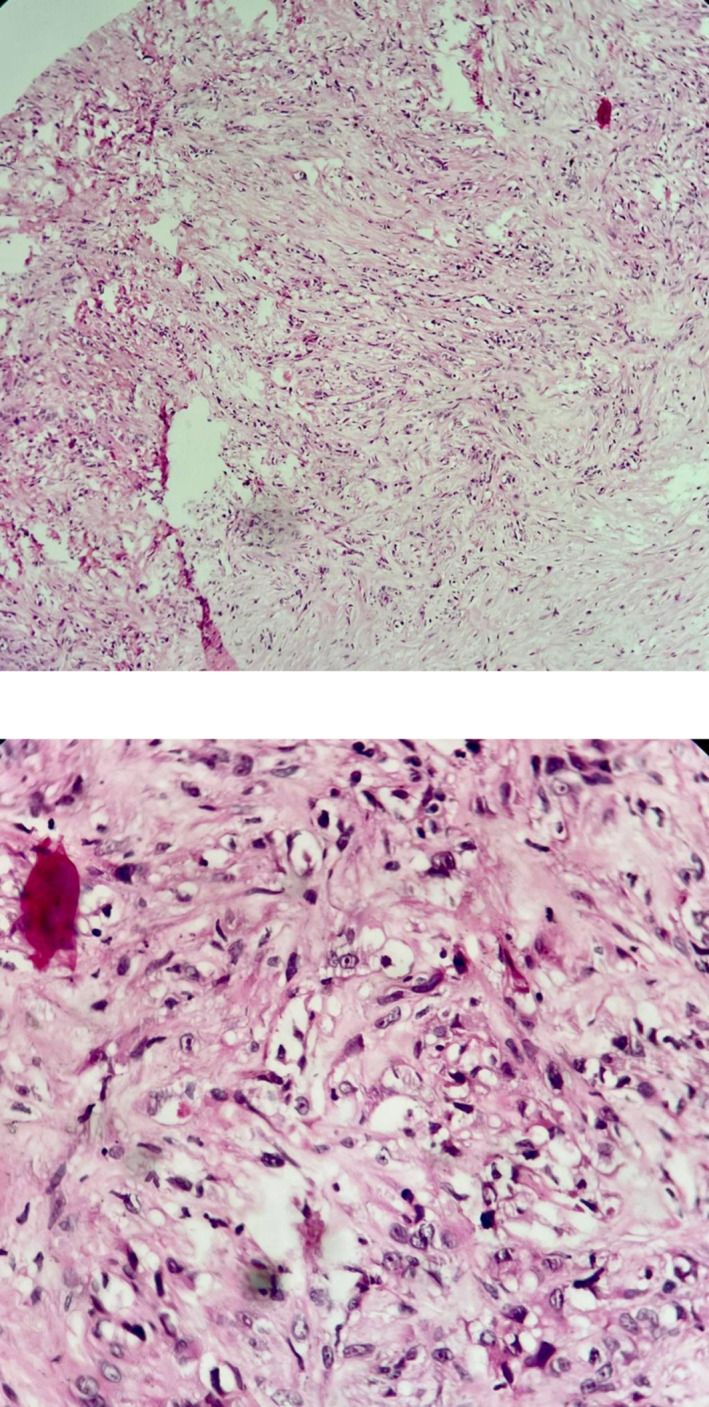
Pleural tissue involved by neoplastic proliferation, atypical epitheloid cells with marked nuclear atypia, mitosis, and vacuolated cytoplasm and focal vascular lumen in a desmoplastic stroma, H&E staining, 40× and 100×, respectively.

The patient had a history of admission to the Department of Thoracic Surgery on April 9th, 2022, due to dyspnea and pleuritic chest pain. On April 17th, 2022, the patient had a chest CT scan, revealing pleural effusion plus pyopneumothorax and consolidation, causing the collapse of the right lower lobe (RLL) in the right hemithorax (Figure 1). The patient was diagnosed with tuberculosis (Tb), which had caused right pleural empyema. The standard four‐drug regimen was followed for treatment, and the patient was discharged. After being discharged, the patient only reported experiencing minor dyspnea, which did not affect her daily functioning. As a result, she was started on anti‐TB medications and did not require further workups.On admission, she declared a 2‐day constipation with abdominal pain but denied any associated hematemesis, fevers, chills, or urinary symptoms. During the first episode 5 years ago, she was evaluated at an outlying health center and diagnosed with peptic ulcer disease. She was managed with omeprazole intermittently. She had no allergies and denied alcohol intake or tobacco use. Her HIV serostatus was negative approximately 1 year prior to presentation. On examination, her general appearance was toxic; she was afebrile, with a heart rate of 120 beats/min, blood pressure of 135/78 mmHg, and respiratory rate of 22/min. The lungs were clear with no rales, rhonchi, wheezes, or rubs. Upon abdominal examination, mild distension was observed with guarding and marked rebound tenderness in the epigastrium. Bowel sounds were absent, and no palpable masses were detected. She was immediately transferred to the general surgical department. On August 10th, 2022, the patient had an abdominal and pelvic CT scan performed, showing pneumoperitoneum, ascites, diffuse mesenteric stranding, and engorged mesenteric vessels. Thereupon, the patient was diagnosed with peritonitis as a result of a perforated peptic ulcer. Also, pleural effusion plus a right pyopneumothorax causing the collapse of the RLL, in the right hemithorax, were reported on the observable lung portions of abdominal and pelvic CT consistent with the prior CT scan (Figure 2). Therefore, a chest tube was inserted along with treatment with broad‐spectrum antibiotics. On August 10th, 2022, the patient was taken to the operating room (OR); after undergoing anesthesia, her abdomen was prepped and draped in sterile fashion; a midline incision was made above and under the umbilicus; upon entrance, 1 L of pus and bile secretions was discharged. On examination, the liver, small intestine, and colons appeared normal. A 2*2 cm perforated peptic ulcer was identified on the anterior wall of the stomach body near the lesser curvature. A biopsy from the edge of the ulcer was obtained, which later indicated no results consistent with neoplasia. The perforated ulcer was repaired, and an omental patch was placed. Finally, the abdomen was irrigated with saline. She was immediately transferred to the Internal Medicine Department's intensive care unit (ICU). On August 13th, 2022, the patient underwent a second look surgery, which showed no postoperational complications. During the ICU stay, clinical workups began to diagnose pulmonary issues. A thoracocentesis sample was sent for lab on August 15th, 2022, which showed hemorrhagic fluid with inflammatory clusters but no atypical cells on microscopy. The patient's condition was not stable throughout the admission; on August 23rd, 2022, despite receiving antibiotics, she was intubated. On August 26th, 2022, a chest tube was inserted to aspirate symptomatic pleural effusion. On August 28th, 2022, a bronchoscopy with bronchoalveolar lavage (BAL) was performed. No abnormal findings were detected. A biopsy and bronchial secretion from the BAL were obtained and sent to the lab. Later, the biopsy indicated chondroid mesenchymal hamartoma as the primary diagnosis. Bronchial secretions were negative for acid‐fast and other microbial pathogens. Also, cytology showed no abnormal findings. A CT scan for the abdomen and pelvis was conducted on September 1st, 2022, which showed no postoperation complications. Also, the right perihilar mass‐like lesion causing the collapse of the RLL, pleural thickening with pleural effusion, and nodular opacities were reported, which were inconsistent with malignancy. On account of mild fever, tachycardia, and increased airway mucus hypersecretion, a bronchoscopy with BAL was performed on September 18th, 2022, showing the same results as the previous bronchoscopy with BAL. Bronchial cultures from BAL were negative for acid‐fast and fungal pathogens. Sputum cultures were positive for *Enterococcus* the first time and *Pseudomonas* the second time. The patient continued to be on broad‐spectrum antibiotics. Blood culture was negative. Often, chest tube secretions were bloody. Further control CTs consistently showed pleural effusion, collapse consolidation, hypodense foci with hyperdensity, and increased pleural thickness. On September 24th, 2022, a transbronchial lung biopsy (TBLB) was performed in the right superior lobar bronchus during flexible bronchoscopy with BAL, indicating hemangioendothelioma plus chronic inflammation along with negative polymerase chain reactions (PCRs) for TB and nontuberculous mycobacterium (NTM) from bronchial secretion collected from BAL. Computed angiography (CTA) ruled out pulmonary embolism. Eventually, the case was presented to the joint meeting of the Pulmonary Department and Department of Thorax Surgery, and a consensus decision was made to perform thoracotomy and invasive pulmonary surgery; consequently, on October 3rd, 2022, the patient underwent surgery. The surgeon reported that due to intrathoracic adhesions causing an unexpandable lung, its resection could not be operated, although a biopsy was obtained and sent for a frozen section. Necrotic tissues were removed as much as possible, together with decortication (Figure 3). According to the frozen section biopsy results, there was a malignant tumor suspected of mesothelioma. On further examination, Clusters of Differentiation 31 (CD31), CD34, vimentin, and epithelial membrane antigen (EMA) were all positive on immunohistochemistry (IHC) along with negative cytokeratin (Figure 4). Microscopy indicated that pleural tissue involved by neoplastic proliferation, atypical epitheloid cells with marked nuclear atypia, mitosis, and vacuolated cytoplasm and focal vascular lumen in a desmoplastic stroma was seen (Figure 5). Taking everything into account, the pathologist reported pulmonary angiosarcoma. On October 15th, 2022, whole body scan and single‐photon emission CT (SPECT/CT) were carried out, demonstrating increased tracer uptake in the 4th–12th right ribs on whole‐body images suggesting local invasion of the tumor and focal involvement with the collapse of the right lung on CT component of the study suggesting PPA. On October 24th, 2022, the patient was started on pazopanib (two 200 mg tablets) on the first day and IV paclitaxel (four 120 mg/m^2^ injectable solutions diluted in 500 ccs of normal saline) on the first, 8th, and 15th day of treatment. Despite being under intensive care, the patient's clinical status continued to deteriorate. The exacerbation of dyspnea necessitated the initiation of bilevel positive airway pressure (BiPAP) to manage the increased respiratory effort. Given the patient's critical state and following consultation with the oncologist, it was concluded that an escalation in chemotherapy dosage was untenable. Consequently, the prognosis was deemed terminal. Eventually, the patient was deceased on November 11th, 2022, due to multiple complications from different organs, recurrent infections, several admissions, and delay of treatment.

## 3 Discussion

PPA is a rare, aggressive, malignant endothelial‐cell tumor [1]. The incidence of PPA is about 0.001%–0.030% [6]. Early diagnosis of the condition is uncommon due to its rarity and low index of suspicion. The literature suggests that the disease is usually diagnosed around the age of 55.9, with men being more commonly affected than women [7]. PPAs have been associated with certain risk factors such as exposure to thorium dioxide, vinyl chloride, radon, Lucite‐ball Plombage, chronic empyema, Tb, Thorotrast, and radiotherapy [8, 9]. Our patient had a history of Tb as the only predisposing risk factor. The most common clinical presentations are hemoptysis, shortness of breath, pneumothorax, and weight loss [8, 10, 11, 12, 13]. It is worth noting that around 20% of cases do not exhibit any symptoms and are usually discovered incidentally or during autopsy [14]. A meta‐analysis of reported cases of PPA revealed a spectrum of chest radiograph findings, ranging from solitary lesions to multiple nodular densities, often accompanied by pleural effusion and pulmonary consolidation. Also, patients with multiple lesions exhibited a more unfavorable prognosis compared to those with solitary lesions. This disparity in prognosis is attributed to the rapid progression observed in cases with multiple lesions, coupled with less effective clinical treatment outcomes [5]. We incidentally found a right perihilar mass‐like lesion causing the collapse of the RLL, pleural thickening with empyema, and nodular opacities in the right hemithorax that were reported on the observable lung portions during an abdominal and pelvic CT scan. CT findings are not specific and can be mistaken for pneumonia, Tb, or diffuse pulmonary hemorrhage [4, 14, 15]. In the study conducted by Ren et al., it was observed that among the 28 cases of PPA, bronchoscopy yielded a positive diagnostic rate of merely 15%, significantly lower than that of invasive pulmonary surgery, which stood at 100%. The infrequency of diagnosis via bronchoscopic biopsy may be attributed to the minimal bronchial involvement by the tumors and the challenges in identifying the atypical pathological characteristics of PPA due to the limited sample size obtained through bronchoscopy [5]. Our results are consistent with these findings as we obtained the definitive diagnosis via invasive pulmonary surgery. The definitive diagnosis is made by performing a biopsy and immunohistochemical analysis. The analysis involves using certain markers, including CD31, CD34, epithelial markers (EMA and cytokeratin), VIII‐related antigen, and FLI‐1. CD31 is relatively specific and highly sensitive out of these markers, as it is detected in almost 90% of cases [16, 17]. Sayan et al. reported strong vimentin expression in neoplastic cells [18]. The histopathological features in our case were compatible with those reported in the literature, and we found CD31, CD34, vimentin, and EMA to be positive. PPAs are rare, and currently, no universally accepted treatment guideline exists [9, 10, 11]. Depending on the stage of the disease, treatment options may include chemotherapy, radiation therapy, surgery, or immunotherapy [15]. Wilson et al. reported a complete response to chemotherapy [19]. In our case, we administered a chemotherapeutic combination of pazopanib and paclitaxel. Regrettably, due to the patient's mortality shortly after the initiation of treatment, we were unable to fully observe the effects of this regimen on the tumor. The reported 5‐year survival rate varies between 16% and 56% [20]. It is important to note that despite the treatment mode, most patients do not survive beyond 1 year from the time of diagnosis [10, 11, 21]. The most prolonged survival period reported was 3 years [15]. Our patient was started on pazopanib, a potent tyrosine kinase inhibitor [21] and antivascular endothelial growth factor (VEGF) [22], and paclitaxel under the supervision of an oncologist. Unfortunately, our patient died due to multiple complications and several admissions; hence, it was not possible to follow up on our treatment for more than a month. Also, a delay in diagnosis perhaps played a role in her death due to a lack of reported data, necessitating this case to be reported. In conclusion, we report a very rare case of PPA found incidentally in a complicated patient. Our intention is to share our diagnostic journey and challenges with one of the rarest pulmonary diseases, not only to contribute to the existing literature but also to encourage the medical community to consider such possibilities when encountered with similar presentations and findings. This could potentially expedite the diagnostic process, allowing for immediate treatment initiation and improving patient survival and longevity prospects. We recommend that the medical community pay close attention to the observable lung portions of abdominal and pelvic CT scans, as these areas are often overlooked [23]. It is essential to conduct further studies to establish a clear diagnostic pathway and treatment approach, especially considering the current lack of definitive guidelines [5]. Our own experience underscores the challenges posed by this diagnostic gap.

In our specific case, the limited biopsy size hindered a swift diagnosis, leading to further time‐consuming resampling.

## Author Contributions

All authors contributed to the study's conception and design. The authors confirm contribution to the paper as follows: study conception and design, data collection, and draft manuscript preparation. All authors reviewed the results and approved the final version of the manuscript.

## Ethics Statement

The authors are accountable for all aspects of the work in ensuring that questions related to the accuracy or integrity of any part of the work are appropriately investigated and resolved. All procedures performed in this study were in accordance with the ethical standards of the institutional and/or national research committee(s) and with the Helsinki Declaration (as revised in 2013). Written informed consent was obtained from the patient for publication of this case report and accompanying images. A copy of the written consent is available for review by the editorial office of this journal.

## Conflicts of Interest

The authors declare no conflicts of interest.

## Data Availability

The data that support the findings of this study are available from the corresponding author upon reasonable request.
